# Plasma Nitridation Effect on *β*-Ga_2_O_3_ Semiconductors

**DOI:** 10.3390/nano13071199

**Published:** 2023-03-28

**Authors:** Sunjae Kim, Minje Kim, Jihyun Kim, Wan Sik Hwang

**Affiliations:** 1 Department of Materials Science and Engineering, Korea Aerospace University, Goyang 10540, Republic of Korea; 2 Department of Smart Air Mobility, Korea Aerospace University, Goyang 10540, Republic of Korea; 3 School of Chemical and Biological Engineering, Seoul National University, Seoul 08826, Republic of Korea; jihyunkim@snu.ac.kr

**Keywords:** Ga_2_O_3_, gallium oxide, plasma nitridation, defect density

## Abstract

The electrical and optoelectronic performance of semiconductor devices are mainly affected by the presence of defects or crystal imperfections in the semiconductor. Oxygen vacancies are one of the most common defects and are known to serve as electron trap sites whose energy levels are below the conduction band (CB) edge for metal oxide semiconductors, including *β*-Ga_2_O_3_. In this study, the effects of plasma nitridation (PN) on polycrystalline *β*-Ga_2_O_3_ thin films are discussed. In detail, the electrical and optical properties of polycrystalline *β*-Ga_2_O_3_ thin films are compared at different PN treatment times. The results show that PN treatment on polycrystalline *β*-Ga_2_O_3_ thin films effectively diminish the electron trap sites. This PN treatment technology could improve the device performance of both electronics and optoelectronics.

## 1. Introduction

An ultra-wide-bandgap (UWB) semiconductor, gallium oxide (Ga_2_O_3_) with a bandgap of ~4.9 eV, has been intensively investigated for applications in power electronics and ultraviolet-C (UVC) photodetectors (PDs) [1,2,3,4,5,6,7,8,9,10]. The Ga_2_O_3_ semiconductor exhibits five different polymorphs designated as *α*, *β*, *γ*, *δ*, and *ε*. Among them, *β*-Ga_2_O_3_ has emerged as the studied phase because of its excellent thermal stability [11]. The electrical and optoelectronic performance of semiconductor devices are mainly affected by the presence of defects or crystal imperfections in the semiconductor. With a metal oxide semiconductor including *β*-Ga_2_O_3_, oxygen vacancies are one of the most common defects and are known to serve as electron trap sites whose energy levels are below the conduction band (CB) edge [12]. Through the electron trap sites below the CB, electrons are easily trapped and de-trapped during device operation, which significantly degrades device performance for both electronics and optoelectronics. For example, the lifetime of photogenerated charge carriers is one of the important parameters in optoelectronics because it can limit the speed of the PD operation. PDs with slow decay times are less suitable for high-speed applications. The slow decay of the photocurrent is attributed to the trapping and de-trapping of the photogenerated electrons through the electron trap sites. Thus, it is important to suppress those trapping and de-trapping processes by eliminating the trap sites. It has been reported that nitrogen (N) doping in a metal oxide can suppress both hole and electron trapping sites [13,14,15,16,17] by passivating or neutralizing the trapping sites. Thus, the N effect on *β*-Ga_2_O_3_ semiconductors should be studied to improve device performance by suppressing the trap density in the semiconductor.

In the current study, we discuss the effects of plasma nitridation (PN) on polycrystalline *β*-Ga_2_O_3_ thin films. In detail, the electrical and optical properties of polycrystalline *β*-Ga_2_O_3_ thin films are compared at different PN treatment times.

## 2. Materials and Methods

### 2.1. Formation of Polycrystalline β-Ga_2_O_3_ and PN Treatment Process

An amorphous Ga_2_O_3_ thin film with a thickness of 100 nm was deposited using a polycrystalline Ga_2_O_3_ target and radio frequency (RF) sputtering [4]. The deposition process was conducted at a pressure of 1.3 mTorr at 70 W with 5 sccm of Ar gas. Nitrogen (N) atoms were introduced into the surface of the amorphous Ga_2_O_3_ surface at a substrate temperature of 370 °C via NH_3_ plasma for either 1 min or 5 min. The PN process was performed at a pressure of 0.3 Torr with 200 sccm of NH_3_ gas at 200 W. The PN-treated amorphous Ga_2_O_3_ turned into *β*-Ga_2_O_3_ after annealing at 900 °C in atmosphere for 1 h.

### 2.2. Characterization of β-Ga_2_O_3_ after PN Process

The optical bandgaps of the polycrystalline *β*-Ga_2_O_3_ thin films at different PN treatment times were extracted using a UV-VIS spectrophotometer (UV-3600 plus, Shimadzu, Kyoto, Japan) via a Tauc plot with a direct bandgap model [18]. The depth profiles of the N atoms and other atoms in the *β*-Ga_2_O_3_ thin films were characterized using secondary ion mass spectrometry (SIMS, IMS 7F, CAMECA, Gennevilliers, France) in a 200 × 200 µm^2^ area using Cs^+^ (6 kV, 10 nA). A photoluminescence (PL) analysis was conducted at 264 nm excitation (Horiba Jobin-Yvon LabRAM HR-800 UV-Visible-NIR, Kyoto, Japan).

### 2.3. Metal-β-Ga_2_O_3_ Semiconductor-Metal (MSM) Photodetector (PD)

An MSM PD was fabricated using a metal electrode made of Ti (5 nm)/TiN (100 nm). The active region of the MSM PD consisted of polycrystalline *β*-Ga_2_O_3_ thin films (100 nm)/SiO_2_ (300 nm) on Si wafer. The transient photo-responses of the *β*-Ga_2_O_3_ MSM PDs with different PN treatment times were compared at 100 V bias voltage under UVC irradiation using a semiconductor parameter analyzer (Keithley 4200-SCS, Tektronix, Beaverton, OR, USA). The irradiation was performed using a specialized light source (Xenon Light Source, GLORIA-X150A, Zolix, Beijing, China) and a monochromator (Omni-λ Monochromator, Omni-λ300i, Zolix, Beijing, China). The wavelength of the UVC light used was 254 nm, and the intensity of the monochromatic light source was 0.63 μW/cm^2^.

## 3. Results and Discussion

**Figure 1:**
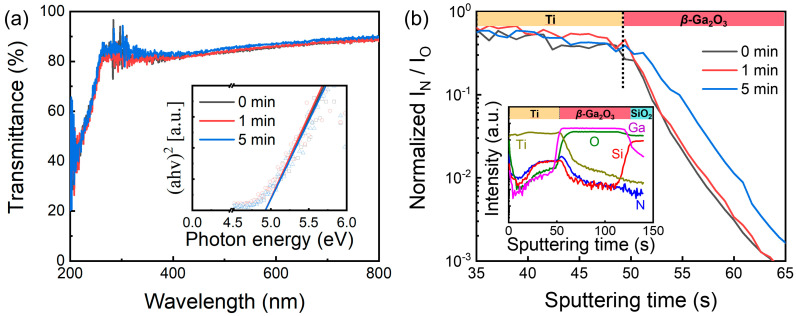
(**a**) Optical transmittance spectra and Tauc−plot and (**b**) normalized ratio of N over O from the SIMS depth profile of the *β*-Ga_2_O_3_ thin film at different PN times. In the inset, SIMS depth profile of intrinsic *β*-Ga_2_O_3_ thin film, which was indistinguishable from the depth profile of Ga, O, and N after 1 and 5 min PN treatment time.

Figure 1a shows the optical transmittance and Tauc plots of the *β*-Ga_2_O_3_ thin film at different PN times. The optical bandgap of each sample was extracted from the linear extrapolation of the inset graph, as shown in Figure 1a. The optical bandgap of the intrinsic *β*-Ga_2_O_3_ thin film was ~4.9 eV, and its value remained almost constant even after PN treatment. This indicated that the PN treatment did not noticeably alter the energy band structure of the *β*-Ga_2_O_3_ semiconductor. The inset of Figure 1b presents a depth profile of the TiN (100 nm)/Ti (5 nm)/intrinsic *β*-Ga_2_O_3_ (100 nm)/SiO_2_ (300 nm)/Si substrate stack showing a uniform Ga and O atomic concentration in the *β*-Ga_2_O_3_ thin film layer. The SIMS depth profiles of Ga, Ti, N, O and Si remained almost constant regardless of PN treatment time. However, the ratio of N/O concentration at different PN times showed that more N was introduced in the polycrystalline *β*-Ga_2_O_3_ thin films at higher PN times. In detail, the ratio of N/O at 1 min PN treatment was indistinguishable from that of the intrinsic *β*-Ga_2_O_3_. However, the ratio of N/O at 5 min PN treatment was much higher compared to that of the intrinsic *β*-Ga_2_O_3_ and *β*-Ga_2_O_3_ at 1 min PN treatment. This indicated that N was noticeably introduced into the *β*-Ga_2_O_3_ in a logical scale after 5 min PN treatment.

**Figure 2:**
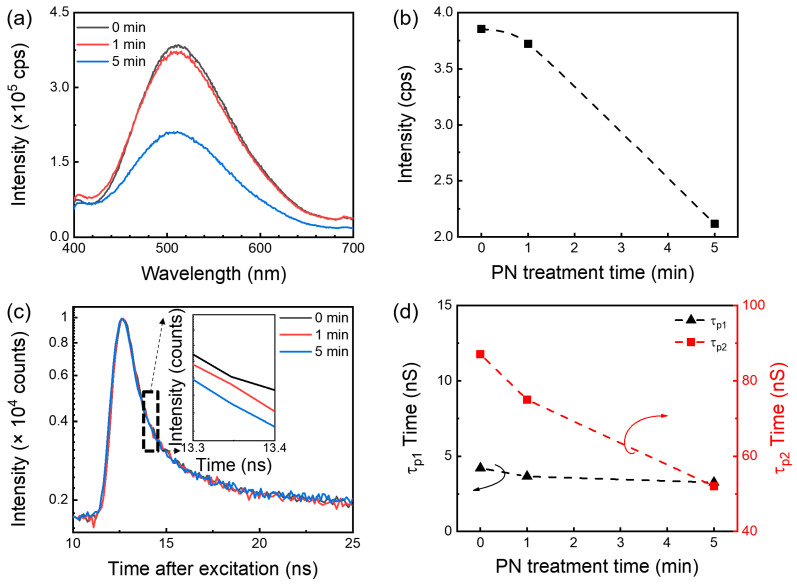
Room temperature PL (**a**) spectra and (**b**) peak intensity as a function of PN time. PL decay (**c**) profile (at 501 nm) and (**d**) time constant of *β*-Ga_2_O_3_ at different PN treatment times. The arrows indicate each axis.

Figure 2a shows the room temperature PL spectrum of the *β*-Ga_2_O_3_ at different PN treatment times. Regardless of the PN treatment, the sputtered *β*-Ga_2_O_3_ thin film exhibited a broad emission band centered around 510 nm. The emission peak in this region presumably originated from the recombination of electrons on donor sites and holes on acceptor sites where the electrons and holes presumably originated from the oxygen vacancy (V_O_) and gallium vacancy (V_Ga_), respectively [19,20,21]. The peak intensities of 510 nm at different PN times are compared in Figure 2b. The results showed that the PL intensity of the *β*-Ga_2_O_3_ with 5 min PN treatment significantly decreased compared to that of the intrinsic *β*-Ga_2_O_3_ and the *β*-Ga_2_O_3_ with 1 min PN treatment. It was assumed that the N effect on the *β*-Ga_2_O_3_ was negligible for the 1 min PN treatment. However, after 5 min PN treatment, a noticeable amount of N atoms was introduced to the *β*-Ga_2_O_3_, as shown in Figure 1b. The introduced N was presumed to effectively suppress the V_O_-related electron trap sites, which resulted in the reduction in PL intensity, as shown in Figure 2a,b.

Furthermore, the PL decay profiles at 510 nm are presented at different PN treatment times and the results are compared in Figure 2c. All of the decay curves were approximated by a sum of two exponential functions (τ_p1_ and τ_p2_). τ_p1_ is an instantaneous response to light and represents a fast response component, while τ_p2_ corresponds to the *β*-Ga_2_O_3_ defect and represents a slow response component. Figure 2d exhibits a comparison of τ_p1_ and τ_p2_ at different PN treatment times. This comparison showed that τ_p1_ remained unchanged while τ_p2_ continued to decrease with PN treatment time. The reduced τ_p2_ with PN treatment was attributed to the suppression of the oxygen vacancies via N doping. It was presumed that the N atoms effectively passivated or neutralized the trapping sites.

**Figure 3:**
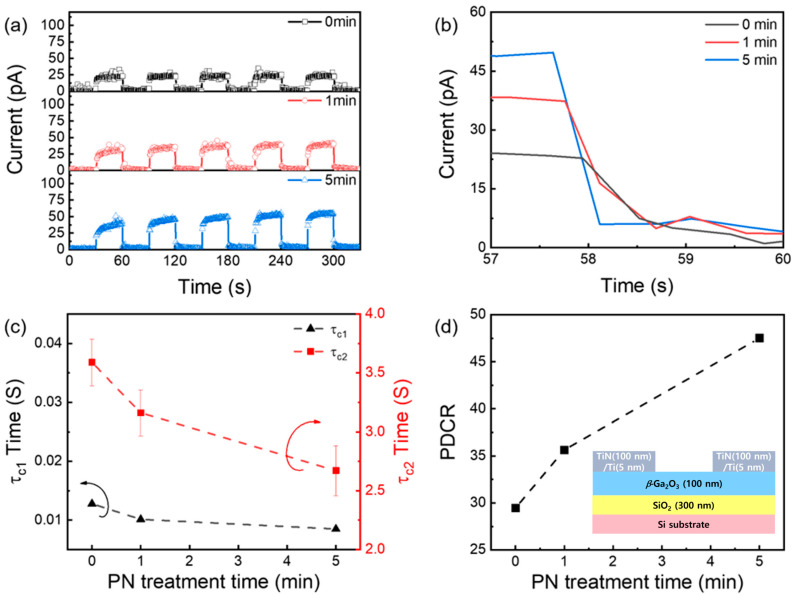
(**a**) Transient photo-response, (**b**) typical photocurrent decay curves, (**c**) two photocurrent decay time constants (τ_c1_ and τ_c2_), and (**d**) PDCR of *β*-Ga_2_O_3_ MSM PDs at different PN treatment times. In the inset, schematic cross-sectional image of the MSM PD. The arrows indicate each axis.

Figure 3a exhibits the transient photo-response of *β*-Ga_2_O_3_ MSM PDs under 254 nm illumination at different PN treatment times. Regardless of the PN treatment, the *β*-Ga_2_O_3_ MSM PDs exhibited a stable repeatability and dark current value as low as 3 pA. A typical photocurrent and its decay time of *β*-Ga_2_O_3_ MSM PDs at different PN treatment times were compared in Figure 3b. The results showed that the photocurrent increased with PN treatment time. The improved photocurrent was attributed to the reduction in the trap density and/or enhancement of carrier mobility via the suppression of trapping sites due to the PN treatment. For a quantitative analysis of the photocurrent decay, the transient curves were fitted using two exponential functions (τ_c1_ and τ_c2_), as shown in Equation (1).

(1) $$I=I_{0}+A\cdot exp(-t/\tau_{c1})+B\cdot exp(-t/\tau_{c2})$$

where $I_{0}$ is the dark current, A and B are constants, and t is the transient time. τ_c1_ and τ_c2_ are the relaxation lifetime decay rate of photo-generated electrons with a fast component (τ_c1_) and a slow component (τ_c2_). The fast component corresponds to the band-to-band transition, while the slow component is attributed to the trapping and de-trapping of photogenerated electrons via defect states. τ_c1_ and τ_c2_ are compared at different PN treatment times in Figure 3c. The results showed that τ_c1_ was less affected by the PN treatment. This was because the band-to-band transition and optical bandgap remained unchanged after PN treatment in these conditions. However, the τ_c2_ values continued to decrease with PN treatment time, indicating that the PN treatment effectively suppressed the trapping and de-trapping processes of the photogenerated electrons via passivating or neutralizing the trapping sites. This is consistent with the results in Figure 2. Finally, photo-to-dark-current (PDCR) of the MSM PD was compared at different PN treatment times, as shown in Figure 3d. The PDCR was defined using Equation (2).

(2) $$PDCR=\frac{I_{p}-I_{d}}{I_{d}}$$

where I_p_ and I_d_ are the photocurrent and dark current of the PD, respectively. The results showed that the PDCR values continued to enhance with PN treatment time. It was noted that the I_d_ remained unchanged with PN treatment, while the I_p_ continued to increase due to the reduction in oxygen vacancies or/and enhancement of carrier mobilities.

**Figure 4:**
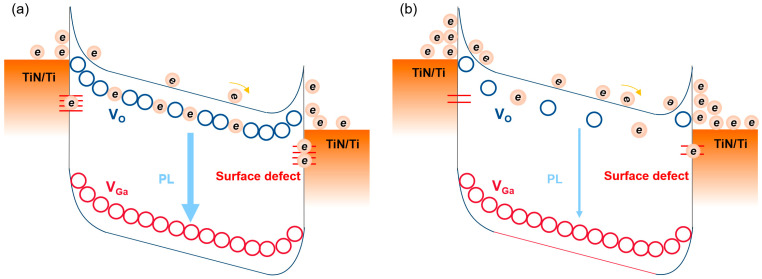
Schematic energy band structure of *β*-Ga_2_O_3_ MSM PD with donor and acceptor levels (**a**) before and (**b**) after PN treatment. Electron trap sties in blue while hole trap sites in red.

Figure 4 shows a schematic drawing of the trap site distribution with donor and acceptor levels before and after PN treatment. The donor and acceptor bands corresponded to the oxygen vacancies and gallium vacancies, respectively. The schematic image demonstrates that the introduced N atoms were able to passivate or neutralize the trap site below the CB and eventually reduce the electron trapping sites. It is believed that the reduced electron trapping sites resulted in a lower PL intensity, fast decay time, and higher PDCR for the *β*-Ga_2_O_3_ MSM PD with a PN treatment of 5 min.

**Figure 5:**
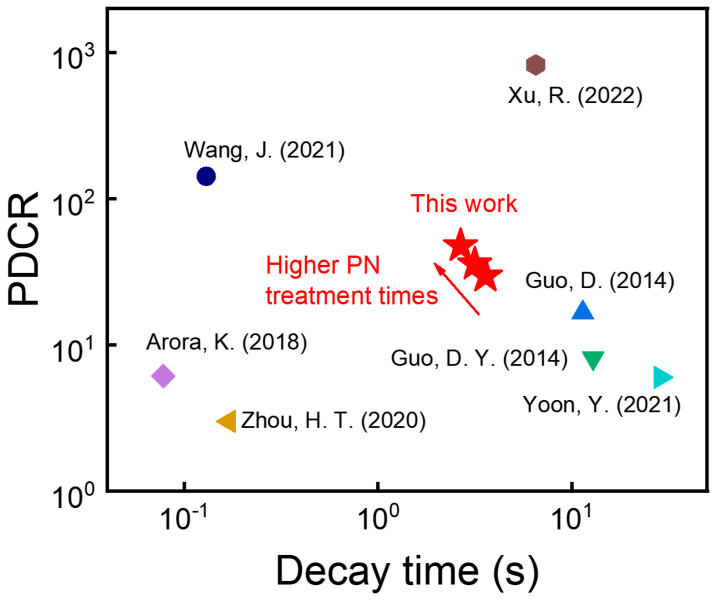
PDCR versus photoresponse decay time of various *β*-Ga_2_O_3_ based MSM PDs. (Yoon, Y. (2021) [4], Wang, J. (2021) [5], Guo, D. (2014) [6], Guo, D. Y. (2014) [7], Arora, K. (2018) [8], Zhou, H. T. (2020) [9], Xu, R. (2022) [10]).

The PDCR and decay time in this work were compared with those of reported *β*-Ga_2_O_3_ based MSM PDs in Figure 5. The results showed that after PN treatment on the polycrystalline *β*-Ga_2_O_3_ thin films, the PDCR value increased but the decay time decreased. It indicated that the device performance of the PDs was enhanced due to the reduction in electron trap sites after PN treatment. This approach could improve the device performance of both electronics and optoelectronics.

## 4. Conclusions

The effects of plasma nitridation (PN) on polycrystalline *β*-Ga_2_O_3_ thin films were investigated. N atoms were introduced in the polycrystalline *β*-Ga_2_O_3_ thin films via NH_3_ plasma. The results showed that the PL intensity of the *β*-Ga_2_O_3_ with 5 min PN treatment significantly decreased compared to that of the intrinsic *β*-Ga_2_O_3_ and the *β*-Ga_2_O_3_ with 1 min PN treatment. In addition, the decay time of both the PL and photocurrent decreased after PN treatment for 5 min, which could be explained by the suppression of the trapping and de-trapping processes of the photogenerated electrons via passivating or neutralizing the trapping sites. It was assumed that the introduced N effectively suppressed the V_O_-related electron trap sites, which resulted in the reduction in the PL intensity, PL decay time, PDCR, and photocurrent decay time. The above results showed that PN treatment on polycrystalline *β*-Ga_2_O_3_ thin films effectively diminished the electron trap sites. This PN treatment technology could improve the device performance of both electronics and optoelectronics.

## Data Availability

The data presented in this study are available on request from the corresponding author.
